# Keratorefractive Lenticule Extraction – KLEx

**DOI:** 10.1055/a-2650-7508

**Published:** 2025-08-21

**Authors:** Marcus Blum, Ramin Khoramnia, Walter Sekundo

**Affiliations:** 1 Augenklinik, HELIOS Klinikum Erfurt, Germany; 2 Augenklinik, Technische Universität Dresden, Germany; 3 Klinik für Augenheilkunde, Universitätsklinikum Gießen und Marburg GmbH, Standort Marburg, Germany

**Keywords:** Refractive Surgery, femtosecond laser, lenticule extraction, KLEx

## Abstract

The clinical use of femtosecond lasers has led to significant progress in a number of surgical procedures in ophthalmology. In the field of corneal refractive surgery, a one-step intrastromal laser procedure without a flap has been established for the first time. This review follows the development of this surgical procedure.

## Introduction: Historical Development

The first attempts at tissue resection at the cornea of the eye to correct myopia were reported many decades ago by the pioneers Barraquer and Ruiz 1 . Using a microkeratome, they removed a layer of tissue from the corneal stroma and referred to it as “in situ keratomileusis”. Their initial results, however, were not convincing due to the mechanical instruments available at the time 2 , 3 .

The development of ultra-short pulsed lasers revolutionised the technical possibilities. The application of chirped pulse amplification (CPA) for the amplification of femtosecond laser pulses was an important step on the way to a technical application and was first described in 1986 by Gérard Mourou and Donna Strickland. In 2018, these physicists were honoured with the Nobel Prize for this invention. Trokel reported the first ablation of corneal tissue by laser in 1983, introducing this technique in refractive surgery 4 . A few years later, a picosecond laser was used in place of the microkeratome to cut an intrastromal lenticule with a constant thickness of 320 µm in a human donor eye 5 . After this was created with a picosecond laser, the flap was lifted and the lenticule was manually removed. In two highly myopic eyes, however, this manipulation led to an irregular surface 6 . This procedure was then also carried out with a femtosecond laser and tested on animal models 7 , 8 .

After the company Intralase, Inc. launched the first femtosecond laser keratome in 2002, individual trials in 2003 for the first time involved extracting laser-cut refractive lenticules from 5 blind or amblyopic eyes 9 , but studies with a larger cohort were never continued. Femtosecond laser keratomes, or simply “femtosecond lasers”, were used for refractive procedures but their use remained limited to cutting the flap, thereby replacing the microkeratome. During the docking process of the laser to the eye, the cornea was maximally applanated, causing a massive increase in intraocular pressure for a short period of time. The actual refractive correction was still performed with the 193 nm excimer laser 10 . However, clinical studies showed a significant improvement in quality compared to mechanical instruments 11 , 12 , 13 .

Around the turn of the millennium, Carl Zeiss Meditec also developed an fs laser with a novel docking system with an interface adapted to the corneal curvature. It was possible to minimise the increase in intraocular pressure 14 , 15 and to allow the patient to fixate on a flashing light. After testing on animal models and docking experiments in humans without actual laser treatment, a prototype femtosecond laser with 200 kHz was initially only marketed as a microkeratome and used in combination with the MEL80 excimer laser 16 .

## Introduction of Femtosecond Lenticule Extraction (FLEx)

**Fig. 1:**
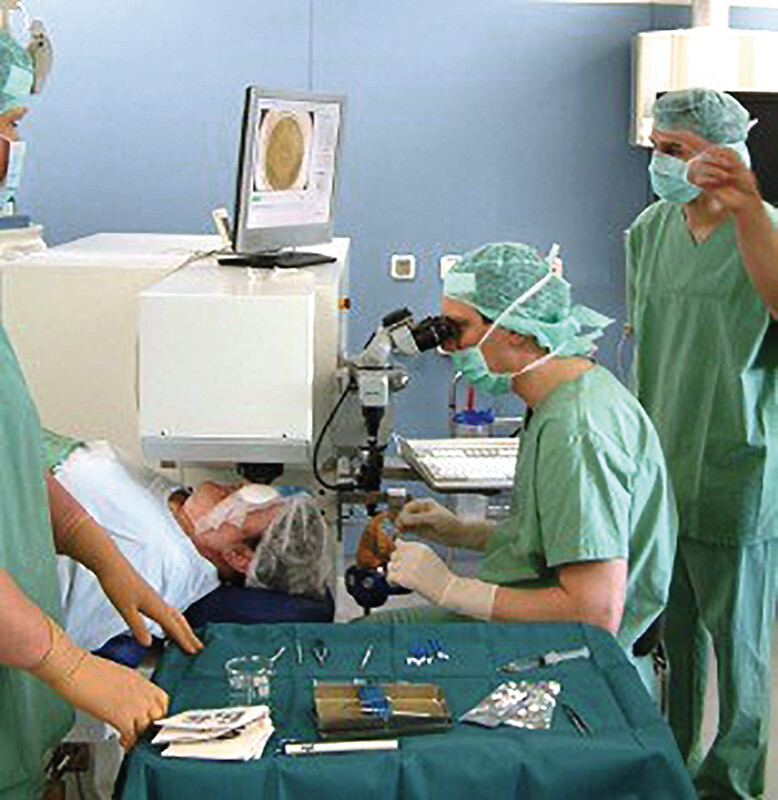
One of the authors of the ‘angular’ prototype of the fs laser performed one of the first lenticule extractions with the Flex technique.

The term “femtosecond lenticule extraction (FLEx)” was coined following studies on animal models and subsequently on blind or amblyopic eyes, for a refractive procedure without the use of an excimer laser ( Fig. 1 ). With a prototype of the laser, not only a flap cut, but a second cut deeper in the stroma was made to remove this small tissue disk as a “lenticule” after lifting the flap. At the Congress of the American Academy of Ophthalmology in Las Vegas, USA, in autumn 2006, Sekundo and Blum presented the first 10 cases of these eyes operated on with a prototype of the Visumax femtosecond laser – the “Horus” laser. Two years later, the first description appeared in the literature 17 .

At this early stage, there were no nomograms. A special calculation was required to determine the geometry and thickness of the lenticules. Postoperative care was the same as for LASIK (laser-assisted in situ keratomileusis), using preservative-free antibiotics, steroids, and artificial tears 4 times a day. The results of the first eyes were followed up for up to 10 years and published 18 , 19 , 20 .

## Development of the “Small Incision Lenticule Extraction” (SMILE)

**Fig. 2:**
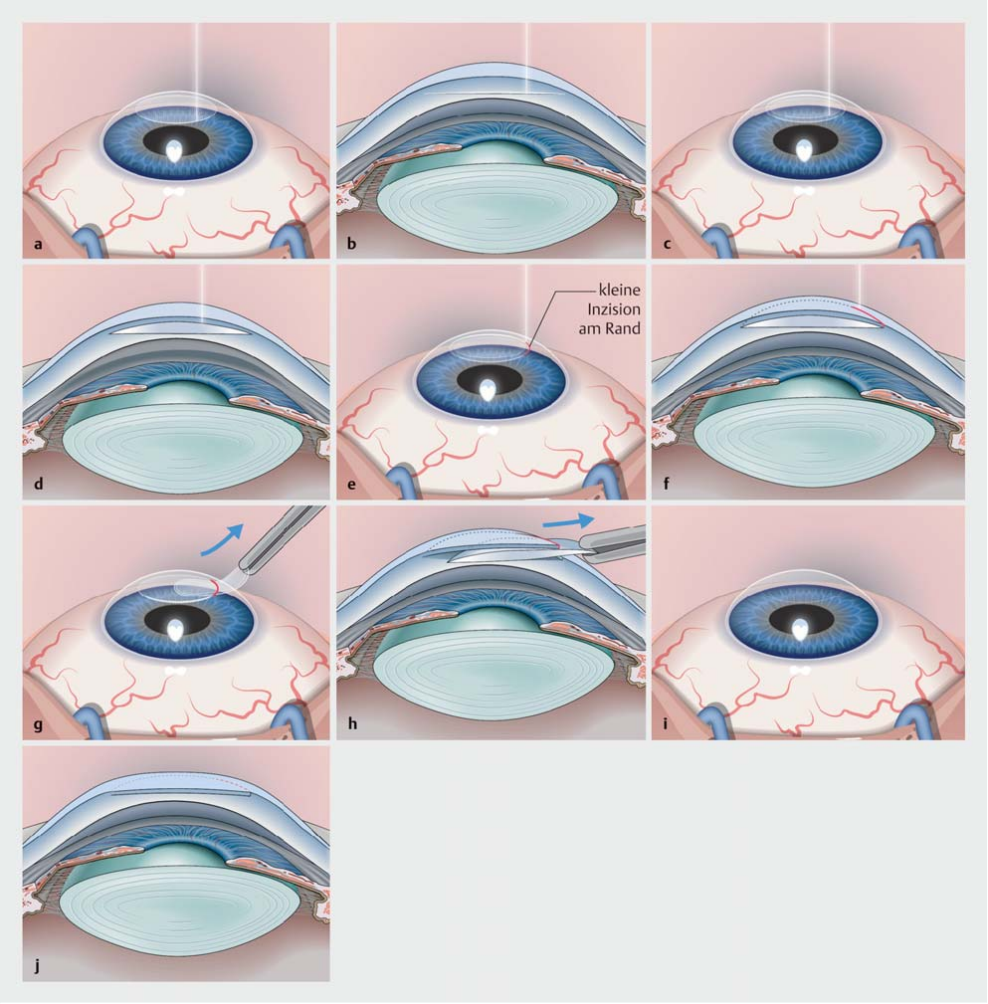
Schematic representation of lenticule extraction (Klex). Source: Blum M, Kunert KS, Sekundo W. Die historische Entwicklung der Small-Incision-Lentikel-Extraktions-OP (SMILE). Klin Monbl Augenheilkd 2017; 234: 117 – 122. doi:10.1055/s-0042-115944 .

**Fig. 3:**
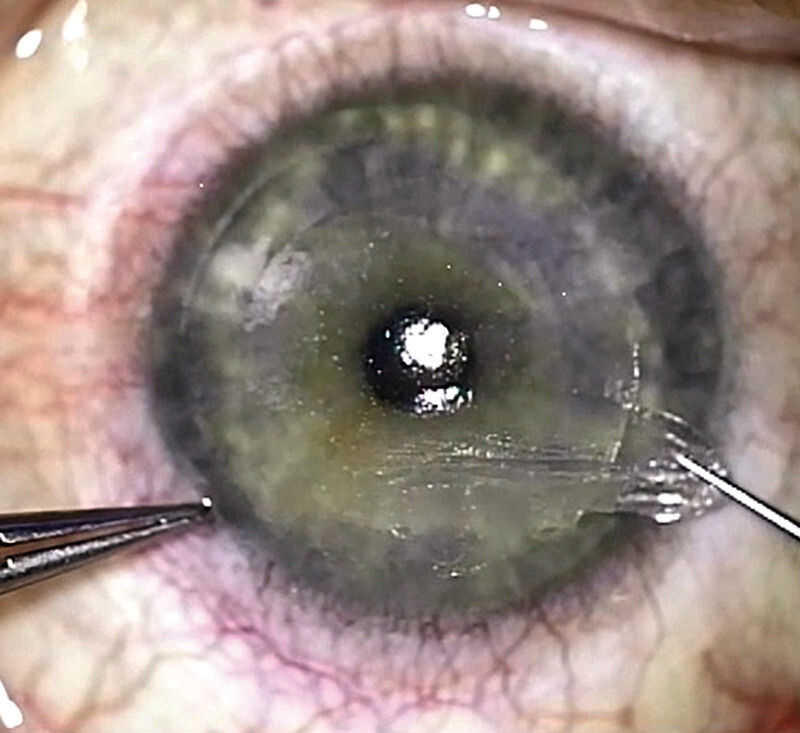
Clinical picture of lenticule extraction.

However, the introduction of the FLEx procedure was intended only as an interim step towards achieving the goal of refractive surgery without lifting a flap. The further development of the surgical procedure was referred to as “Small Incision Lenticule Extraction” and became internationally known under the abbreviation “SMILE”. After creating the fs-laser cutting planes, the anterior and posterior surfaces of the lenticule are carefully separated using a small spatula through a 2 – 3 mm incision, and then the lenticule is extracted through the incision ( Fig. 2 ). It was no longer necessary to lift a flap and the former flap now became a further ‘cap’ firmly connected to the corneal stroma. In 2009, this SMILE method was first demonstrated in a video at the “Video Cataratta Refrattiva” in Milan, but it took 3 years for the first reviewed work to be printed 21 . In contrast to the current procedure, 2 small incisions of 4 mm each were made opposite each other in this study, but a “proof of concept” was clearly established for the first time and the results encouraged further users to test the new technique 22 , 23 , 24 . The energy settings of the “old” 200 kHz laser prototype were optimised, and the scanning or cutting direction of the fs laser was modified, as a significant impact on the quality of the cuts was demonstrated. Rupal Shah was the first surgeon to use only a single incision and systematically developed the minimal incision size used in surgery today. This is generally between 2 and 3 mm for myopic SMILE ( Fig. 3 ).

A new 500 kHz laser was introduced by Carl Zeiss Meditec AG, thus increasing the speed of lenticule cutting 25 , 26 . Although good refractive results were already achieved in the first study, which were not far removed from the results of the LASIK, a lively discussion about the advantages and disadvantages of the method began in parallel. With good contrast sensitivity outcomes but higher-order aberrations, a slower visual recovery was observed compared to LASIK 27 . Data on safety and complications were already convincing around 10 years ago in large patient cohorts from other study centres 28 , 29 . From 2010/11 onwards, the technique spread internationally, and by now well over 10 million eyes worldwide have been treated using lenticule extraction. Stability data are available over 5 and 10 years 30 , 31 , 32 . The main reasons for the adaptation of flapless technology are

- better mechanical stability 33 and
- less loss of sensitivity in the first postoperative months and thus fewer problems in the postoperative “dry eye” 34

As demonstrated by the most recent prospective multicentre study, with the latest generation devices – such as the 2 MHz Visumax 800 (Carl Zeiss Meditec, Jena) laser – the disadvantage of slower visual recovery has also disappeared 35 . As expected, the basic final outcome of the SMILE operation is not dependent on whether the Visumax 500 or the newer Visumax 800 is used, because both lasers have the same optics. However, not only surgeons, but also patients prefer the Visumax 800 laser, presumably because of its speed: myopia treatment with a zone of 6.5 mm takes only 9 seconds with the Visumax 800 instead of 24 seconds 36 . This reduces the probability of a suction loss. In addition, the Visumax 800 is currently the only approved laser for the treatment of hyperopia and hyperopic astigmatism (see below).

## Revision

Although the precision of lenticule extraction is convincing, revision is still required in isolated cases. In principle, enhancement procedures after SMILE can be performed without difficulty. Surface ablation using mitomycin C largely preserves corneal stability but is regarded by patients as very “unpleasant” due to the long and painful rehabilitation period.

The flap variants in the form of a standard femtoflap with the same parameters as well as in the form of a single sidecut with transition zone (Circle procedure) are clearly preferred by patients due to the short rehabilitation phase. With these flap variants, however, the advantages offered by the originally applied SMILE method are not retained. Nevertheless, the Circle procedure remains the method of choice for many practitioners when it comes to enhancement treatments 37 .

## Other Laser Manufacturers

Continuous improvements in technology (e.g. optimisation of laser parameters) and laser sources with higher pulse frequency significantly shortened treatment times. Computer-assisted centration and compensation of cyclorotation were developed. Due to the proven advantages for postoperative sicca syndrome and good stability, the technique of lenticule extraction, which had initially only been developed by the company Carl Zeiss Meditec AG with the Visumax fs laser, was taken up by several companies with their respective fs laser systems and also introduced to the market. However, the term “SMILE” is trademark protected, creating the need for new, company-neutral nomenclature for the procedure. In the context of product neutrality, the term “lenticule extraction” or the abbreviation KLEx is used for keratorefractive lenticule extraction 38 . Nevertheless, individual companies have introduced their own brand names for the same process with their respective fs lasers.

## Z8 (Ziemer)

Ziemer equipped the Femto-LGVZ8 laser with an application called “Clear”, which stands for “Corneal Lenticule Extraction for Advanced Refractive Correction”. The rollable mobile system, already introduced to the market for other applications (e.g. flap cutting in LASIK, cataract surgery), operates with low-energy pulses in the nanojoule range and a high repetition rate (20 MHz). The precision of the laser cut has been demonstrated 39 . This laser also has a centration option and compensation of cyclorotation. During docking, a shallow cone is drawn in and the applanation of the cornea causes a marked increase in intraocular pressure. In a spiral grid, overlapping laser spots are placed in the tissue. It is possible to carry out a “guided” lenticule extraction via 2 tunnels, in which the front and back of the lenticule are prepared separately. The device has an intraoperative OCT. The first studies on the efficiency of the laser in human use are available in the literature 40 .

## Atos (Schwind eye-tech solutions)

Another fs laser approved for lenticule extraction is the Atos laser by Schwind. “Atos Smartsight”, a microsection technology for minimally invasive removal of a corneal lenticule has been CE certified since 2020. The precision of the lenticule cuts was demonstrated in animal studies 41 . This device has an eye tracking system with pupil detection and can compensate for the cyclorotation. The deviceʼs pulse rate reaches up to 4 MHz while using low energy of up to 75 nJ in a quasi-telecentric optical configuration. The eye is fixed with a lower vacuum (~ 250 mmHg) and, as with ZEISS, the cornea is docked with a curved surface (20 mm). Lenticule geometry does not contain a minimum lenticule thickness and is refined with a refractive transition zone. The first scientific publications on the use of this laser in patients are also available in the literature 42 , 43 , 44 .

## Elita (Johnson&Johnson)

With the fs-Laser Elita, Johnson&Johnson launched a lenticule extraction system under the name “Smooth Incision Lenticule Keratomileusis (Silk)”. The interface for docking the patientʼs eye applanates the corneal surface flat, as with the Ziemer device, and is therefore likely to cause significant short-term increases in intraocular pressure. The laser has pulse durations of 100 – 250 fs with a pulse repetition rate of 10 MHz. The maximum pulse energy is 200 nJ with a laser spot spacing of 1 µm. According to the company, this is the only device that produces biconvex laser-cut lenticules.

Additionally, Elita takes a different approach to cutting the lenticule by using a radial rather than a spiral laser scan. The first encouraging clinical results have been published by two Indian working groups 45 , 46 .

Comparing the currently available technologies, differences can be observed in the docking systems (flat applanation vs. curved interface), lenticule geometry, cutting patterns, and applied pulse energies ( Table 1 ). The importance of the level of energy input into the corneal tissue has been demonstrated 47 . In the next few years, there will have to be a further comparative, scientific analysis of the extent to which this parameter can further increase the quality of the results of lenticule extraction.

**Table:** **Table 1** Different parameters of commercially available lasers with lenticule extraction module.

Manufacturer	ZEISS	Schwind	Ziemer	Johnson&Johnson
Name of the fs laser	VISUMAX 800	ATOS	Z8 NEO	Elita
Module for KLEx	SMILE pro	SmartSight	CLEAR	SILK
Treatment of Myopia and Astigmatism	sph to − 10 D cyl to − 5 D	sph to − 10 D cyl to − 5 D	sph to − 10 D cyl to − 5 D	sph to − 12 D cyl to − 8 D
Treatment of Hyperopia and Astigmatism	sph to + 7 D cyl to + 4 D	None	None	None
Laser Repetition Rate	2 MHz	4 MHz	< 11 MHz	10 MHz
Laser Treatment Time	Myopia < 10 s hyperopia < 13 s	Myopia > 30 s	Myopia > 30 s	Myopia ~ 15 s
Centration	Guided Docking System	Guided Docking System	Correction after Docking	Correction after Docking
Cyclotorsion	Automatic Cyclotorsion Correction	Automatic Cyclotorsion Correction	Marking required	Marking required
Docking system	Curved with suction	Curved with suction	Applanation	Applanation
Integrated microscope	Yes	Yes	No	No
Integrated slit lamp	Yes	No	No	No
Other uses of lasers	Flap, CIRCLE, Keratoplasty, ICR	Flap	Flap, keratoplasty, ICR, pockets, FLACS	Flap

Other, innovative light sources could also have an impact: All of the above-mentioned lasers have wavelengths in the near-infrared range. Physically, a significantly lower energy input into the tissue with shorter wavelengths in the ultraviolet spectrum would be possible. Corresponding lasers had already been tested previously 48 , 49 .

## Hyperopia Correction

Until now, only the Visumax 800 femtosecond laser from Carl Zeiss Meditec AG has also been approved for the correction of hyperopia and hyperopic astigmatism in almost the entire world (FDA approval in the USA is still pending). While lenticule extraction to correct myopic astigmatism quickly proved successful, the early results of hyperopia correction were sobering as regression persistently clouded the result 50 . By changing the lenticule geometry and enlarging the optical zone, significantly better results were achieved 51 , 52 . A working group in Nepal researched the diameter of the optical zone and the optimal centration of the hyperopic lenticule 53 , 54 . The same working group then also tested the new laser profiles with improved results 55 .

These preliminary studies provided important insights which were then incorporated into a multicentre study on the treatment of hyperopia. Patients with hyperopia or hyperopia with astigmatism were treated in 9 clinics across 6 countries. They had a predicted postoperative keratometry of less than 51 D and a preoperative distance visual acuity of 0.8 or better. This multicentre study has now been published and the results of the 374 eyes are available 56 .

At the 12-month follow-up, 81% of the eyes were within ± 0.5 D and 93% of the eyes were within ± 1.00 D of the target refraction. Only 1.2% of the eyes had lost 2 or more lines of vision. The refraction results remained gratifyingly stable over the 12-month follow-up period. Only a mild regression was observed. An initial overcorrection to − 0.17 D (± 0.53 D) was reversed to − 0.02 D (± 0.57 D) in the first 3 months. After 12 months, the final result was + 0.12 D.

**Fig. 4:**
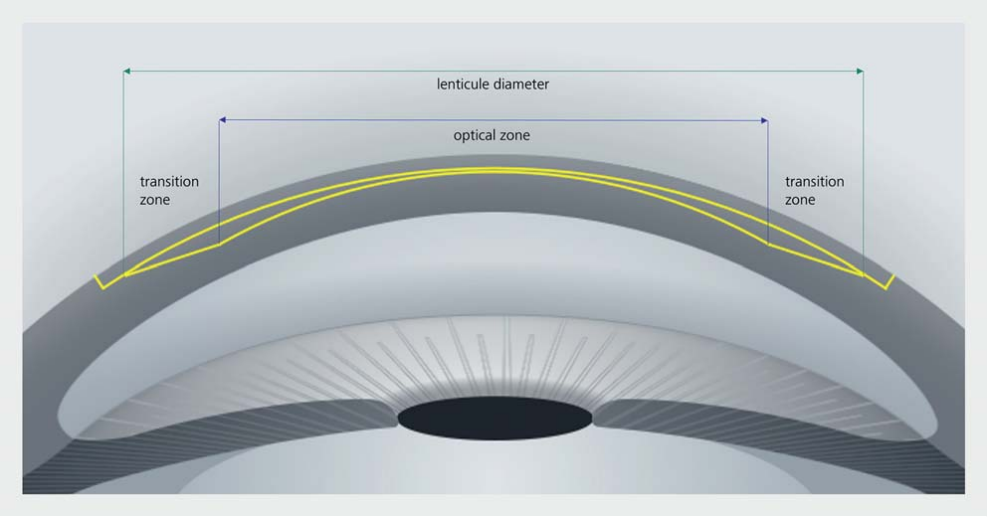
Schematic representation of lenticule geometry in hyperopic lenticule extraction. Source: Carl Zeiss Meditec AG, Jena.

Compared to the treatment of myopic eyes, two additional parameters must be considered and carefully planned before surgery in the treatment of hyperopia:

Firstly, the surgeon must define the minimum central thickness of the lenticule and secondly, the transition zone must be defined (see Fig. 4 ). These two parameters and their effect on the refractive procedure are discussed in more detail below.

## The Central Lenticule Thickness

The term “central lenticule thickness” describes the thinnest part of the tissue to be removed during hyperopia correction. In the case of hyperopia, this is located centrally in the optical axis. A rupture or defect in the lenticule would therefore have very negative consequences for postoperative visual acuity and must be prevented. The minimum lenticule thickness can therefore be set to at least 25 µm, but can also be increased up to a thickness of 60 µm. It should be noted that while increasing the central lenticule thickness does improve safety during lenticule preparation, it also increases the volume of tissue to be removed, thereby causing a greater weakening of corneal stability due to the refractive procedure.

## The Transition Zone

The transition zone is necessary in hyperopic lenticule extraction to counteract regression. The transition zone on the device can be adjusted by the user from 2.00 mm to 3.85 mm. The contact glass of the treatment pack is available in 3 sizes, i.e. 3 different diameters. Due to the larger transition zone compared to myopia, the use of an “M” contact glass is almost always necessary; only in the case of a small optical zone can an “S” contact glass also be used. The possible cap diameter is between 7.85 mm and 9.7 mm depending on the treatment pack size. The thickness of the cap is adjustable between 100 µm and 160 µm.

These two additional parameters of lenticule extraction result in changes to the surgical planning process for hyperopia:

- The larger lenticule diameter with the transition zone leads to a slightly longer laser time compared to myopic lenticule extraction. Longer laser time with a larger contact glass increases the risk of suction loss. This is compensated for by the significant increase in speed on the Visumax 800. The average laser time for a hyperopic lenticule with a 7 mm zone is 12 s.
- The larger lenticule diameter must also be taken into account when selecting surgical instruments. A spatula that is too short during the dissection of the cutting planes can lead to incomplete lenticule preparation and carries the risk of incomplete extraction.

The treatment range for hyperopic correction ranges spherically from + 0.15 D to + 7.0 D, for astigmatism cylinders from 0.0 D to 4.0 D with an axis of 0 – 180° can be used. However, not every hyperopic eye is suitable for lenticule extraction. The main limitation lies in the fact that hyperopic eyes often have a small corneal diameter. For example, a minimum white-to-white distance (WTW) of 11.7 mm is required for the use of an M contact glass. For this purpose, the angle kappa must also be added, since the farsighted generally fix nasally from the centre of the pupil. Example: A hyperopic eye with a κ = 0.6 mm must have a minimum white-to-white of at least 12.3 mm. In spite of the rather wide approval range, the authors (as with the LASIK surgery) believe that corrections above + 4.0 D should be avoided. In the multicentre study, it was already apparent that the more the target correction exceeded the value of + 3.0 D, the more severe the regression 56 .

## Use of the Lenticule

Since treatment of hyperopia is not aimed at flattening but at steepening the curvature of the cornea, there is currently discussion not only of the subtractive method with extraction of corneal tissue, but also of additional methods with the transmission of corneal lenticules (“re-shaping of the cornea”).

The implantation of donor or autologous tissue in a corneal pocket can be used for both refractive and therapeutic purposes. This basic idea goes back to the pioneer J. I. Barraquer, who already described “keratophakia” in the middle of the last century 57 . Because of the technologies available at the time, it took until the beginning of this century to implement the idea. T. Seiler was the first to use tissue treated with excimer lasers 58 . The introduction of the femtosecond laser with lenticule extraction today results in the availability of a large number of fresh lenticules from healthy corneal stroma. K. Pradhan was the first to implant a − 10.0 D lenticule into the corneal pocket of an aphakic eye generated by fs-laser to reduce the refractive deficit 59 . In doing so, he achieved only a partial effect because the tissue addition caused a change in curvature not only towards the anterior but also towards the posterior corneal surface. The first transplantation of a toric lenticule to correct severe astigmatism following a complicated LASIK operation with mixed-up laser data in Germany was published by the Marburg working group in 2016 60 .

S. Ganesh from India performed this technique on a large number of eyes, using both frozen and fresh lenticules. In a 5-year study in 42 eyes with a 140 µm pocket, 71% of the eyes were within 1 D of target refraction. A circular trephination of the Bowmanʼs layer helped in undercorrected eyes by reducing the residual hyperopia by around 2 D 61 .

Several Chinese working groups published their results, some with a cap thickness of 100 µm 62 , 63 . X. Zhou from Shanghai also calculated the first formula for predicting lenticule thickness: (LAC) = 1,224 × (LRP) − 0.063. Here, LAC is the refractive correction and LRP is the lenticule refractive power. She corrected hyperopia with a concomitant astigmatism of up to 2 D.

J. Hjortdalʼs working group devoted itself to correcting high astigmatism. In 2019, Damgaard published the first ex vivo study on autologous lenticule rotation 64 .

P. Stodulka was able to implement these results clinically 65 . Lenticule rotation can also be combined with excimer laser correction to address more complex ametropias: While Shang et al. demonstrated this with high myopic astigmatism 66 , Sekundo et al. showed the 2-year results with high astigmatism and hyperopia 67 . The treatment of presbyopia by means of a central 1 mm mini-transplant, which is inserted into a femto-pocket, was also described by S. Jacobs. She called this technique Pearl 68 .

Despite a more complex surgical technique and the potential risk of rejection (legally, it is a corneal transplant or autorotation keratoplasty), additive techniques are often the only way to correct these complexities in high ametropias. Fortunately, the procedure is nearly completely reversible if the result is unsatisfactory.

## Conclusion

The surgical technique of flapless lenticule extraction using fs laser (KLEx) for correction of myopic astigmatism has proven successful. Further technical refinement is expected, as the market environment is highly competitive. With the same technique, correction of hyperopia is now also available.

By introducing lenticule extraction, healthy, refractively exactly cut corneal tissue in the form of a tissue lens is available (Latin “lenticula”). In addition to the previous subtractive methods of refractive surgery, additive and rotation techniques represent a therapeutic option for cases that have not hitherto been treatable.
