# Supplementary material for: Keratorefractive Lenticule Extraction – KLEx
Source: Klin Monbl Augenheilkd. 2025 Aug 21;242(8):795–802. doi: 10.1055/a-2650-7508 (PMC12370370; doi:10.1055/a-2650-7508)
Supplement: Related texts

# Research article: Keratorefraktive Lentikel-Extraktion (KLEx) – Stand des Verfahrens

**Keywords:** refraktive Chirurgie, Femtosekundenlaser, Lentikel, Extraktion, KLEx

## Abstract

Mit der Einführung von Femtosekundenlasern in die klinische Augenheilkunde wurden deutliche Fortschritte bei vielen chirurgischen Verfahren erreicht. Im Bereich der refraktiven Hornhautchirurgie wurde es mit der Technik der Lentikel-Extraktion erstmals möglich, die Korrektur im Stroma der Hornhaut mit nur einem Laser ohne Anheben eines Flaps durchzuführen. Der Übersichtsartikel zeigt die schrittweise Entwicklung zu diesem mittlerweile international etabliertem Verfahren.

## Einleitung: die historische Entwicklung

Über erste Versuche einer Geweberesektion an der Hornhaut des Auges zur Korrektur der Myopie wurde schon vor vielen Jahrzehnten von den Pionieren Barraquer und Ruiz berichtet 1 . Mittels Mikrokeratom entfernten sie eine Gewebeschicht aus dem Hornhautstroma und sprachen dabei von einer „In-situ-Keratomileusis“. Ihre ersten Ergebnisse waren aufgrund der damals zur Verfügung stehenden mechanischen Instrumente allerdings nicht überzeugend 2 , 3 .

Durch die Entwicklung von ultrakurz gepulsten Lasern wurden die technischen Möglichkeiten revolutioniert. Die Anwendung der Chirped-Pulse-Amplification (CPA) für die Verstärkung von Femtosekunden-Laserimpulsen war ein wesentlicher Schritt auf dem Weg zur technischen Anwendung und wurde erstmals 1986 von Gérard Mourou und Donna Strickland beschrieben. 2018 wurden diese Physiker mit dem Nobelpreis für diese Erfindung geehrt. Von der ersten Ablation von Hornhautgewebe mittels eines Lasers berichtete Trokel 1983 und führte damit diese Technik in die refraktive Chirurgie ein 4 . Wenige Jahre später wurde ein Pikosekundenlaser anstelle des Mikrokeratoms genutzt, um in einem humanen Spenderauge einen intrastromalen Lentikel mit einer konstanten Dicke von 320 µm zu schneiden 5 . Nachdem dieser mit einem Pikosekundenlaser angelegt worden war, wurde der Flap angehoben und der Lentikel manuell entfernt. In 2 hochmyopen Augen führte diese Manipulation allerdings zu einer unregelmäßigen Oberfläche 6 . Dieses Verfahren wurde dann auch mit einem Femtosekundenlaser (fs-Laser) durchgeführt und an Tiermodellen erprobt 7 , 8 .

Nachdem im Jahr 2002 die Firma Intralase, Inc. das erste Femtosekunden-Laserkeratom auf den Markt gebracht hatte, wurden in Einzelversuchen 2003 erstmals an 5 blinden oder amblyopen Augen mit Laser geschnittene refraktive Lentikel extrahiert 9 , jedoch nie Versuche mit einer größeren Kohorte fortgesetzt. Femtosekunden-Laserkeratome, oder kurz „Femtosekundenlaser“, wurden für refraktive Eingriffe genutzt, blieben in ihrem Einsatz jedoch darauf beschränkt, den Flap zu schneiden und damit das Mikrokeratom zu ersetzen. Beim Andockprozess des Lasers an das Auge wurde die Hornhaut maximal applaniert und dadurch für kurze Zeit eine massive Steigerung des Intraokulardrucks verursacht. Die tatsächliche refraktive Korrektur wurde weiterhin mit dem 193 nm Excimerlaser durchgeführt 10 . Klinische Studien zeigten aber eine deutliche Qualitätsverbesserung gegenüber den mechanischen Instrumenten 11 , 12 , 13 .

Auch die Firma Carl Zeiss Meditec entwickelte um die Jahrtausendwende einen fs-Laser mit einem neuartigen Andocksystem mit einem der Hornhautkrümmung angepassten Inferface. Es gelang die intraokulare Druckerhöhung zu minimieren 14 , 15 und dem Patienten die Fixation eines Blinklichtes zu ermöglichen. Nach der Erprobung an Tiermodellen und Andockversuchen am Menschen ohne tatsächliche Laserbehandlung wurde ein Prototyp eines Femtosekundenlasers mit 200 kHz zunächst nur als Mikrokeratom auf den Markt gebracht und in Kombination mit dem MEL80 Excimerlaser eingesetzt 16 .

## Einführung der Femtosekunden-Lentikel-Extraktion (FLEx)

Der Begriff der „Femtosekunden-Lentikel-Extraktion (FLEx)“ wurde nach Studien an Tiermodellen und nachfolgend an blinden oder amblyopen Augen für eine refraktive Operation ohne Einsatz eines Excimerlasers geprägt ( Abb. 1 ). Dabei wurde mit einem Prototyp des Lasers nicht nur ein Flapschnitt, sondern ein 2., tiefer im Stroma gelegener Schnitt angelegt, um nach dem Anheben des Flaps diese kleine Gewebescheibe als „Lentikel“ zu entfernen. Beim Kongress der American Academy of Ophthalmology in Las Vegas/USA wurden von Sekundo und Blum im Herbst 2006 die ersten 10 Fälle dieser mit einem Prototyp des Visumax Femtosekundenlasers – dem „Horus“-Laser – operierten Augen vorgestellt. Zwei Jahre später erschien die Erstbeschreibung in der Literatur 17 .

**Abb. 1:** Einer der Autoren am „eckigen“ Prototyp des fs-Lasers bei einer der ersten Lentikel-Extraktionen mit FLEx-Technik.

Zu diesem frühen Zeitpunkt gab es noch keinerlei Nomogramme. Eine spezielle Berechnung war erforderlich, um die Geometrie und die Dicke der Lentikel zu bestimmen. Die Nachbehandlung erfolgte wie bei einer LASIK durch konservierungsmittelfreie Antibiose, Steroide und 4×/d Tränenersatzmittel. Die Ergebnisse der ersten Augen wurden bis zu 10 Jahre nachverfolgt und veröffentlicht 18 19 20 .

## Entwicklung der „Small Incision Lenticule-Extraction“ (SMILE)

Die Einführung der Flex-Operation war jedoch nur als ein Zwischenschritt gedacht, um das Ziel einer refraktiven Chirurgie ohne Anheben des Flaps zu realisieren. Die Weiterentwicklung des OP-Verfahrens wurde als „Small Incision Lenticule Extraction“ bezeichnet und wurde unter der Abkürzung „SMILE“ international bekannt. Nach Anlegen der fs-Laser Schnittebenen werden mit einem kleinen Spatel durch eine 2 – 3 mm Inzision die Vorder- und Rückfläche des Lentikels freipräpariert und dieser dann über die Inzision extrahiert ( Abb. 2 ). Das Anheben eines Flaps war nicht mehr erforderlich und aus dem ehemaligen Flap wurde jetzt ein weiter fest mit dem Hornhautstroma verbundener „Cap“. Im Jahr 2009 wurde dieses SMILE-Verfahren bei der „Video Cataratta Refrattiva“ Mailand erstmals im Video vorgeführt, aber es dauerte 3 Jahre, bis die erste begutachtete Arbeit gedruckt vorlag 21 . Im Gegensatz zum heute üblichen Vorgehen wurden in dieser Studie noch 2 gegenüberliegende kleine Inzisionen von jeweils 4 mm angelegt, es war aber erstmals eindeutig ein „Proof of Concept“ erfolgt und die Ergebnisse ermutigten weitere Anwender die neue Technik zu erproben und zu testen 22 , 23 , 24 . Die Energie-Einstellungen des “alten“ 200 kHz Laser-Prototypen wurden optimiert, und die Scan- oder Schneiderichtung des fs-Lasers wurde modifiziert, da ein deutlicher Einfluss auf die Schnittqualität nachgewiesen wurde. Rupal Shah nutzte als erste Operateurin nur eine Inzision und erarbeitete systematisch die minimale Inzisionsgröße, mit der heute operiert wird. Diese liegt in der Regel zwischen 2 und 3 mm bei myoper SMILE ( Abb. 3 ).

**Abb. 2:** Schematische Darstellung der Lentikel-Extraktion (KLEx). Quelle: Blum M, Kunert KS, Sekundo W. Die historische Entwicklung der Small-Incision-Lentikel-Extraktions-OP (SMILE). Klin Monbl Augenheilkd 2017; 234: 117 – 122. doi:10.1055/s-0042-115944

**Abb. 3:** Klinisches Bild der Lentikel-Extraktion.

Von der Firma Carl Zeiss Meditec AG wurde ein neuer 500-kHz-Laser vorgestellt und damit die Schnelligkeit des Lentikelschnitts erhöht 25 , 26 . Obwohl bereits in der ersten Studie gute refraktive Ergebnisse erzielt wurden, die nicht weit von den Ergebnissen der LASIK entfernt lagen, begann parallel eine lebhafte Diskussion über die Vor- und Nachteile des Verfahrens. Mit guten Werten beim Kontrastsehen und den höheren Aberrationen zeigte sich eine langsamere Erholung des Visus gegenüber der LASIK 27 . Die Daten zur Sicherheit und den Komplikationen waren auch in großen Patientenkohorten anderer Studienzentren bereits vor rund 10 Jahren überzeugend 28 , 29 . Ab 2010/11 breitete sich die Technik international aus, und mittlerweile sind weltweit weit über 10 Millionen Augen mittels Lentikel-Extraktion behandelt worden. Es liegen Stabilitätsdaten über 5 und 10 Jahre vor 30 , 31 , 32 . Als Hauptgründe für die Adaptation der flaplosen Technik sind vor allem zu nennen

- Bessere mechanische Stabilität 33 und
- geringerer Sensibilitätsverlust in den ersten postoperativen Monaten und damit geringere Probleme beim postoperativen „trockenen Auge“ 34 .

Wie die jüngste prospektive Multicenterstudie nachweist, ist mit den Geräten der letzten Generation, wie dem 2 MHz Visumax 800 (Carl Zeiss Meditec, Jena) Laser auch der Nachteil der langsameren Visuserholung verschwunden 35 . Wie zu erwarten ist das grundsätzliche Endergebnis der SMILE-Operation nicht davon abhängig, ob der Visumax 500 oder der neuere Visumax 800 genutzt wird, denn beide Laser verfügen über eine identische Optik. Allerdings ziehen nicht nur Operateure, sondern auch Patienten den Visumax 800 Laser vor, vermutlich aufgrund seiner Geschwindigkeit: eine Myopie-Behandlung bei 6,5-mm-Zone dauert mit dem Visumax 800 nur noch 9 s statt bisher 24 s 36 . Damit ist die Wahrscheinlichkeit eines Ansaugverlustes („suction loss“) reduziert. Außerdem ist der Visumax 800 gegenwärtig der einzige zugelassene Laser zur Behandlung von Hyperopie und hyperopen Astigmatismus (s. u.).

## Revisionen

Auch wenn die Präzision der refraktiven Korrektur bei der Lentikel-Extraktion überzeugend ist, bleibt doch im Einzelfall die Notwendigkeit einer Revision. Grundsätzlich sind Nachkorrekturen nach SMILE problemlos durchzuführen. Eine Oberflächen-Ablation unter Anwendung von Mitomycin C erhält weitgehend die Hornhautstabilität, wird aber von den Patienten aufgrund der langen und schmerzhaften Rehabilitationszeit als sehr „unangenehm“ empfunden.

Die Flap-Varianten in Form eines Standard-Femtoflaps mit gleichen Parametern als auch in Form eines alleinigen Sidecuts mit Übergangszone (Circle-Prozedur) werden aufgrund der kurzen Rehabilitationsphase von den Patienten eindeutig bevorzugt. Mit diesen Flap-Varianten bleiben die Vorteile, die das ursprünglich angewendete SMILE-Verfahren bietet, jedoch nicht erhalten. Dennoch ist das Circle-Verfahren für viele Anwender die Methode der Wahl für Nachkorrekturen 37 .

## Weitere Laserhersteller

Kontinuierliche Verbesserungen der Technik (z. B. Optimierung der Laserparameter) und Laserquellen mit höherer Pulsfrequenz verkürzten die Behandlungszeiten signifikant. Eine computerassistierte Zentrierung und eine Kompensation der Zyklorotation wurden entwickelt. Aufgrund der nachgewiesenen Vorteile für das postoperative Sicca-Syndrom und der guten Stabilität wurde die Technik der Lentikel-Extraktion, die zunächst nur von der Firma Carl Zeiss Meditec AG mit dem Visumax fs-Laser entwickelt worden war, von mehreren Firmen mit ihren jeweiligen fs-Lasersystemen aufgegriffen und ebenfalls in den Markt eingeführt. Der Begriff „SMILE“ ist jedoch markenrechtlich geschützt, sodass die Notwendigkeit einer neuen „firmenneutralen“ Nomenklatur für das Verfahren bestand. Im Rahmen der Produktneutralität wird der Begriff der „Lentikel-Extraktion“ oder die Abkürzung KLEx für Keratorefraktive Lentikel-Extraktion genutzt 38 . Trotzdem haben einzelne Firmen eigene Markennamen für das gleiche Verfahren mit ihren jeweiligen fs-Lasern eingeführt.

## Z8 (Ziemer)

Ziemer hat den Femto-LGVZ8-Laser mit einer Applikation, die kurz „Clear“ für „Corneal Lenticule Extraction for Advanced Refractive Correction“ heißt, ausgestattet. Das für andere Applikationen (z. B. Flapschnitt bei der LASIK, Kataraktchirurgie) bereits in den Markt eingeführte und durch Rollen mobile System arbeitet mit Niedrigenergiepulsen im nJ-Bereich und hoher Repetitionsrate (20 MHz). Die Präzision der Schnittführung mit dem Laser wurde nachgewiesen 39 . Auch dieser Laser hat eine Zentrierungsoption und Kompensation der Zyklorotation. Beim Andocken wird mit einem flachen Konus angesaugt und durch die Applanation der Hornhaut eine deutliche Erhöhung des intraokularen Drucks verursacht. In einem spiralförmigen Raster werden überlappende Laserspots im Gewebe platziert. Es besteht die Möglichkeit, über 2 Tunnel eine „geführte“ Lentikelextraktion durchzuführen, bei der jeweils die Vor- und Rückseite des Lentikels präpariert werden. Das Gerät hat ein intraoperatives OCT. Erste Arbeiten zur Effizienz des Lasers beim Einsatz am Menschen liegen in der Literatur vor 40 .

## Atos (Schwind eye-tech-solutions)

Ein weiterer für die Lentikel-Extraktion zugelassener fs-Laser ist der Atos-Laser der Firma Schwind. Mit „Atos Smartsight“ ist eine Mikroschnitt-Technologie zur minimalinvasiven Entfernung eines kornealen Lentikels seit 2020 CE-zertifiziert. Die Präzision der Lentikelschnitte wurde im Tierversuch nachgewiesen 41 . Dieses Gerät hat ein Eye-Tracking-System mit Pupillenerkennung und kann die Zyklorotation kompensieren. Die Pulsrate des Gerätes erreicht bis zu 4 MHz unter Verwendung niedriger Energie bis zu 75 nJ in einer quasi-telezentrischen optischen Anordnung. Das Auge wird mit geringerem Vakuum (~ 250 mmHg) fixiert und wie bei ZEISS erfolgt das Andocken an die Hornhaut mit einer gekrümmten Oberfläche (20 mm). Die Lentikelgeometrie enthält keine „minimum lenticule thickness“ und ist mit einer refraktiven Übergangszone verfeinert. Auch zu diesem Laser sind die ersten wissenschaftlichen Veröffentlichungen über die Anwendung an Patienten in der Literatur zugänglich 42 , 43 , 44 .

## Elita (Johnson&Johnson)

Mit dem fs-Laser Elita hat auch Johnson&Johnson unter dem Namen „Smooth Incision Lenticule Keratomileusis (Silk)“ ein System zur Lentikel-Extraktion auf den Markt gebracht. Das Interface zum Andocken des Patientenauges applaniert bei diesem Gerät die Hornhautoberfläche wie bei Ziemer flach und dürfte damit auch kurzfristig deutliche Erhöhungen des Augendrucks verursachen. Der Laser hat Pulsdauern von 100 – 250 fs mit einer Pulsrepetitionsrate von 10 MHz. Die maximale Pulsenergie liegt bei 200 nJ mit einem Laserspotabstand von 1 µm Als einziges Gerät sind die gelaserten Lentikel nach Angaben der Firma biconvex.

Außerdem geht Elita einen anderen Weg beim Schneiden des Lentikels, indem der Laserscan nicht spiralförmig, sondern radiär verläuft. Die ersten ermutigenden klinischen Ergebnisse wurden von zwei indischen Arbeitsgruppen publiziert 45 , 46 .

Vergleicht man die derzeit vorliegenden Technologien, kann eine unterschiedliche Vorgehensweise bei den Andocksystemen (flache Applanation vs. gekrümmtes Interface), bei der Lentikelgeometrie, den Schnittmustern und bei den eingetragenen Pulsenergien festgestellt werden (siehe Tab. 1 ). Die Bedeutung der Höhe des Energieeintrages in das korneale Gewebe ist nachgewiesen 47 . Es wird in den nächsten Jahren eine weitere vergleichende, wissenschaftliche Aufarbeitung geben müssen, inwiefern dieser Parameter die Ergebnisqualität der Lentikel-Extraktion weiter steigern kann.

**Table:** **Tab. 1** Unterschiedliche Parameter der auf dem Markt befindlichen Laser mit Modul zur Lentikel-Extraktion.

Name des fs-Laser	VISUMAX 800	ATOS	Z8 NEO	Elita
Modul für KLEx	SMILE pro	SmartSight	CLEAR	SILK
Behandlung Myopie und Astigmatismus	sph bis − 10 dpt cyl bis − 5 dpt	sph bis − 10 dpt cyl bis − 5 dpt	sph bis − 10 dpt cyl bis − 5 dpt	sph bis − 12 dpt cyl bis − 8 dpt
Behandlung Hyperopie und Astigmatismus	sph bis + 7 dpt cyl bis + 4 dpt	keine	keine	keine
Laser Repetitionsrate	2 MHz	4 MHz	< 11 MHz	10 MHz
Laser Behandlungszeit	Myopie < 10 s Hyperopie < 13 s	Myopie > 30 s	Myopie > 30 s	Myopie ~ 15 s
Zentrierung	geführtes Andocksystem	geführtes Andocksystem	Korrektur nach Docking	Korrektur nach Docking
Cyclotorsion	automatische Cyclotorsionskorrektur	automatische Cyclotorsionskorrektur	Markierung erforderlich	Markierung erforderlich
Andocksystem	gebogen mit Saugung	gebogen mit Saugung	Applanation	Applanation
integriertes Mikroskop	ja	ja	nein	nein
integrierte Spaltlampe	ja	nein	nein	nein
weitere Anwendungen des Lasers	Flap, CIRCLE, Keratoplastik, ICR	Flap	Flap, Keratoplastik, ICR, pockets, FLACS	Flap

Auch weitere, innovative Lichtquellen könnten Einfluss haben: Alle genannten Laser haben Wellenlängen im Nah-Infrarotbereich. Physikalisch wäre ein deutlich geringerer Energieeintrag in das Gewebe mit kürzeren Wellenlängen im ultravioletten Spektrum möglich. Entsprechende Laser waren früher bereits in der Testung 48 , 49 .

## Korrektur der Hyperopie

Bisher wurde nur der Femtosekundenlaser Visumax 800 der Firma Carl Zeiss Meditec AG auch zur Korrektur der Hyperopie und hyperopen Astigmatismus in nahezu der ganzen Welt zugelassen (die FDA-Zulassung in den USA steht noch aus). Während sich die Lentikel-Extraktion zur Korrektur des myopen Astigmatismus schnell bewährt hat, waren die frühen Ergebnisse der Hyperopie-Korrektur ernüchternd, da die Regression das Ergebnis nachhaltig trübte 50 . Durch eine Veränderung der Lentikelgeometrie und eine Vergrößerung der optischen Zone wurden deutlich bessere Ergebnisse erzielt 51 , 52 . Eine Arbeitsgruppe in Nepal beschäftigte sich mit dem Durchmesser der optischen Zone und der optimalen Zentrierung des hyperopen Lentikels 53 , 54 . Die gleiche Arbeitsgruppe testete dann auch die neuen Laser-Profile mit verbesserten Ergebnissen 55 .

Durch diese Vorstudien wurden wichtige Erkenntnisse gewonnen, die dann in eine multizentrische Studie zur Behandlung der Hyperopie eingeflossen sind. In 9 Kliniken aus 6 Ländern wurden Patienten mit Hyperopie oder Hyperopie mit Astigmatismus behandelt, die eine vorausberechnete postoperative Keratometrie von < 51 dpt und einen präoperativen Fernvisus von 0,8 oder besser hatten. Diese multizentrische Studie ist mittlerweile publiziert und die Ergebnisse der insgesamt 374 Augen liegen vor 56 .

Bei der 12 Monatskontrolle lagen 81% der Augen innerhalb ± 0,5 dpt und 93% der Augen innerhalb ± 1,00 dpt der Zielrefraktion. Nur 1,2% der Augen hatten 2 oder mehr Zeilen an Visus verloren. Die Refraktionsergebnisse blieben über den Kontrollzeitraum von 12 Monaten erfreulich stabil. Nur eine leichte Regression war zu beobachten. Eine anfängliche Überkorrektur auf − 0,17 dpt (± 0,53 dpt) bildete sich in den ersten 3 Monaten auf − 0,02 dpt (± 0,57 dpt) zurück. Nach 12 Monaten lag das Endergebnis bei + 0,12 dpt.

Gegenüber der Behandlung von myopen Augen müssen präoperativ 2 zusätzliche Parameter bei der Behandlung der Hyperopie bedacht und genau eingeplant werden:

Zum einen muss die minimale zentrale Dicke des Lentikels vom Operateur definiert werden. Zum zweiten muss die Übergangszone („transition zone“) definiert werden ( Abb. 4 ). Auf diese beiden Parameter und ihren Einfluss auf den refraktiven Eingriff soll im Folgenden genauer eingegangen werden.

**Abb. 4:** Schematische Darstellung der Lentikelgeometrie bei hyperoper Lentikel-Extraktion.

## Die zentrale Lentikeldicke

Der Begriff „zentrale Lentikeldicke“ beschreibt die dünnste Stelle des bei der Hyperopie-Korrektur zu entfernenden Gewebes. Diese liegt bei Hyperopen zentral in der optischen Achse. Ein Riss oder Defekt im Lentikel hätte daher sehr negative Folgen für die postoperative Sehschärfe und muss verhindert werden. Die minimale Lentikeldicke ist daher auf mindestens 25 µm einstellbar, aber auch bis zu einer Dicke von 60 µm zu erhöhen. Zu Bedenken ist, dass eine Vergrößerung der zentralen Lentikeldicke die Sicherheit bei der Präparation des Lentikels zwar erhöht, aber auch das Volumen des zu entfernenden Gewebelentikels erhöht und damit die Schwächung der Hornhautstabilität durch den refraktiven Eingriff größer wird.

## Die Übergangszone

Die Übergangszone ist bei hyperoper Lentikel-Extraktion notwendig, um einer Regression entgegenzuwirken. Am Gerät ist die Übergangszone von 2,00 mm bis 3,85 mm durch den Anwender einstellbar. Das Kontaktglas des „treatment-packs“ ist in 3 Größen, also 3 unterschiedlichen Durchmessern, verfügbar. Durch die gegenüber der Myopie größere Übergangszone ist fast immer die Verwendung eines „M“-Kontaktglases erforderlich, nur bei kleiner optischer Zone kann auch ein „S“-Kontaktglas verwendet werden. Der mögliche Cap-Durchmesser liegt zwischen 7,85 mm bis 9,7 mm in Abhängigkeit von der Treatment Pack Größe. Die Dicke des Caps ist zwischen 100 µm bis 160 µm einstellbar.

Durch diese zwei zusätzlichen Parameter der Lentikel-Extraktion ergeben sich bei der Hyperopie Veränderungen in der Ablaufplanung der OP:

- Der größere Lentikeldurchmesser mit der Übergangszone führt zu einer etwas längeren Laserzeit gegenüber der myopen Lentikel-Extraktion. Längere Laserzeit mit einem größeren Kontaktglas erhöht das Risiko eines Saugverlustes („suction-loss“). Dies wird beim Visumax 800 durch die deutliche Erhöhung der Geschwindigkeit ausgeglichen. Die durchschnittliche Laserzeit für einen hyperopen Lentikel mit einer 7-mm-Zone liegt bei 12 s.
- Der größere Lentikeldurchmesser muss auch bei der Auswahl der OP-Instrumente berücksichtigt werden. Ein zu kurzer Spatel bei der Dissektion der Schnittebenen kann zu den unvollständigen Lentikel-Präparationen führen und birgt die Gefahr der unvollständigen Extraktion.

Der Behandlungsbereich für die hyperope Korrektur reicht sphärisch von + 0,15 dpt bis + 7,0 dpt, beim Astigmatismus können Zylinder von 0,0 dpt bis 4,0 dpt mit Achse von 0 – 180° therapiert werden. Dennoch, nicht jedes hyperope Auge eignet sich für eine Lentikel-Extraktion. Die Haupteinschränkung liegt in der Tatsache, dass vor allem hyperope Augen nicht selten einen kleinen Hornhautdurchmesser haben. So ist für die Verwendung eines M-Kontaktglases ein Mindest-Weiß-zu-Weiß-Abstand (WzW) von 11,7 mm erforderlich. Dazu muss außerdem der Winkel Kappa addiert werden, da die Weitsichtigen in der Regel nasal von der Pupillenmitte fixieren. Beispiel: Ein hyperopes Auge mit einem κ = 0,6 mm muss über einen WzW von mindestens 12,3 mm verfügen. Trotz des recht weiten Zulassungsbereiches sollten nach der Meinung der Autoren (genau wie bei der LASIK-OP) möglichst keine Korrekturen über + 4,0 dpt vorgenommen werden. Bereits in der Multicenterstudie zeichnete sich ab, dass je weiter die Zielkorrektur den Wert von + 3,0 dpt überschritt, umso stärker war die Regression 56 .

## Nutzung des Lentikels

Da bei einer Therapie der Hyperopie nicht eine Abflachung, sondern eine Aufsteilung der Hornhaut das Ziel ist, wird aktuell nicht nur das subtraktive Verfahren mit Extraktion von Hornhautgewebe, sondern auch über additive Verfahren mit der Übertragung von Hornhautlentikeln diskutiert („re-shaping of the cornea“).

Die Implantation von fremdem oder eigenem Gewebe in einer Hornhauttasche kann dabei sowohl für refraktive als auch für therapeutische Zwecke genutzt werden. Auch diese grundsätzliche Idee geht auf den Pionier J. I. Barraquer zurück, der bereits Mitte des letzten Jahrhunderts die sogenannte „Keratophakie“ beschrieb 57 . Aufgrund der damals zur Verfügung stehenden Technologien dauerte es bis zu den Anfängen dieses Jahrhunderts bis zur Umsetzung des Gedankens. T. Seiler setzte erstmals mit Excimerlasern behandeltes Gewebe ein 58 . Die Einführung des Femtosekunden-Lasers mit der Lentikel-Extraktion führt heute dazu, dass eine große Menge an frischen Lentikeln aus gesundem Hornhautstroma zur Verfügung steht. K. Pradhan war der erste, der einen − 10,0 dpt Lentikel in die mittels eines fs-Lasers erzeugte Hornhauttasche eines aphaken Auges implantierte, um das Refraktionsdefizit zu reduzieren 59 . Dabei erzielte er nur einen Teileffekt, weil die Gewebeaddition eine Krümmungsänderung nicht nur nach vorne, sondern auch Richtung Hornhautrückfläche wirkte. Die erste Transplantation eines torischen Lentikels zur Wiederherstellung eines extremen Astigmatismus nach komplikativer LASIK-OP mit verwechselten Laserdaten in Deutschland publizierte die Marburger Arbeitsgruppe 2016 60 .

S. Ganesh aus Indien führte diese Technik an einer großen Anzahl von Augen durch, wobei er sowohl tiefgefrorene als auch frische Lentikel nutzte. In einer 5-Jahres-Studie an 42 Augen mit 140-µm-Tasche lagen 71% der Augen innerhalb von 1 dpt um die Zielrefraktion. Eine zirkuläre Antrepanation der Bowman-Schicht half bei unterkorrigierten Augen, indem sie die Resthyperopie um rund 2 dpt verringerte 61 .

Mehrere chinesische Arbeitsgruppen veröffentlichten ihre Ergebnisse, teilweise auch mit einer Cap-Dicke von 100 µm 62 , 63 . X. Zhou aus Shanghai berechnete auch die erste Formel für die Vorhersage der Lentikelstärke: (LAC) = 1,224 × (LRP) − 0,063. Dabei ist LAC die refraktive Korrektur und LRP die Lentikelbrechkraft. Damit korrigierte sie Hyperopien mit einem Begleitastigmatismus von bis zu 2 dpt.

Der Korrektur eines hohen Astigmatismus widmete sich die Arbeitsgruppe von J. Hjortdal. 2019 publizierte Damgaard die erste Ex-vivo-Studie zur autologen Lentikelrotation 64 .

Diese Ergebnisse konnte P. Stodulka klinisch umsetzen 65 . Eine Lentikelrotation kann auch mit einer Excimerlaser-Korrektur kombiniert werden, um komplexere Ametropien anzugehen: Während Shang et al. dies bei hohem myopem Astigmatismus demonstrierten 66 zeigte Sekundo et al die 2-Jahresergebnisse bei hohem Astigmatismus und Hyperopie 67 . Auch die Behandlung der Presbyopie mittels eines zentralen 1-mm-Minitransplantates, das in eine Femto-Tasche eingesetzt wird, wurde von S. Jacobs beschrieben. Sie nannte diese Technik Pearl 68 .

Trotz einer komplexeren chirurgischen Technik und der potenziellen Gefahr einer Abstoßung (es handelt sich rechtlich um eine Hornhauttransplantation bzw. Autorotation-Keratoplastik) sind additive Techniken bei hohen Ametropien oft die einzige Möglichkeit diese komplexen Augen zu korrigieren. Erfreulicherweise ist der Eingriff bei nicht zufriedenstellendem Ergebnis nahezu komplett reversibel.

## Zusammenfassung

Die chirurgische Technik der flaplosen Lentikel-Extraktion mittels fs-Laser (KLEx) zur Korrektur des myopen Astigmatismus hat sich bewährt. Eine weitere technische Perfektionierung ist in Aussicht, da eine ernstzunehmende kompetitive Marktsituation besteht. Mit der gleichen Technik ist inzwischen auch eine Korrektur der Hyperopie gegeben.

Durch die Einführung der Lentikel-Extraktion steht gesundes, refraktiv exakt geschnittenes korneales Gewebe in der Form einer Gewebelinse zur Verfügung (lateinisch „lenticula“). Neben den bisherigen subtraktiven Methoden der refraktiven Chirurgie stellen additive und Rotationstechniken eine therapeutische Option für bisher nicht behandelbare Fälle dar.
